# Medical Needs Extraction for Breast Cancer Patients from Question and Answer Services: Natural Language Processing-Based Approach

**DOI:** 10.2196/32005

**Published:** 2021-10-28

**Authors:** Masaru Kamba, Masae Manabe, Shoko Wakamiya, Shuntaro Yada, Eiji Aramaki, Satomi Odani, Isao Miyashiro

**Affiliations:** 1 Division of Information Science Graduate School of Science and Technology Nara Institute of Science and Technology Nara Japan; 2 Cancer Control Center Osaka International Cancer Institute Osaka Japan

**Keywords:** natural language processing, internet use, patient generated health data, neoplasms

## Abstract

**Background:**

A large number of patient narratives are available on various web services. As for web question and answer services, patient questions often relate to medical needs, and we expect these questions to provide clues for a better understanding of patients’ medical needs.

**Objective:**

This study aimed to extract patients’ needs and classify them into thematic categories. Clarifying patient needs is the first step in solving social issues that patients with cancer encounter.

**Methods:**

For this study, we used patient question texts containing the key phrase “breast cancer,“ available at the Yahoo! Japan question and answer service, Yahoo! Chiebukuro, which contains over 60,000 questions on cancer. First, we converted the question text into a vector representation. Next, the relevance between patient needs and existing cancer needs categories was calculated based on cosine similarity.

**Results:**

The proportion of correct classifications in our proposed method was approximately 70%. Considering the results of classifying questions, we found the variation and the number of needs.

**Conclusions:**

We created 3 corpora to classify the problems of patients with cancer. The proposed method was able to classify the problems considering the question text. Moreover, as an application example, the question text that included the side effect signaling of drugs and the unmet needs of cancer patients could be extracted. Revealing these needs is important to fulfill the medical needs of patients with cancer.

## Introduction

### Background

Patients with cancer have many medical needs. These needs are diverse and not necessarily communicated to doctors, nurses, and other medical staff. A database of their problems is needed to determine which patients experience problems or have unmet needs and to what extent.

Such a database does exist in Japan, the “cancer problem classification” (CPC), and is maintained by the Shizuoka Cancer Center. It was created by collecting and categorizing the claims of cancer survivors through a nationwide survey into 4 categories. The CPC has systematized the worries and burdens of patients with cancer surveyed through telephone consultations and other means, with 7855 participants in 2003 and 4054 in 2013 [1]. However, this process was conducted manually by experts, and making a new one would be costly and time-consuming. With the recent exponential growth of the internet, a vast number of illness-related problems have already been accumulated in Japan [2], where blogs are actively written. As of July 2021, TOBYO [3] has the largest collection of diaries and blogs in Japan dedicated to battling diseases (approximately 63,000 of such diaries and blogs), covering some 1500 conditions. Among them, the number of blogs on breast cancer, the treatment of which tends to be prolonged, is particularly large, accounting for over 10% (approximately 6900) of blogs. Furthermore, Yahoo! Japan’s question and answer (YJQA) service, commonly called Chiebukuro [4], is one of Japan's leading question and answer (QA) services, containing approximately 60,000 questions that include the key phrase “breast cancer.” Thus, a vast archive of patient claims has already been created on the internet.

In this context, many recent studies have utilized accumulated information [5-9]. For example, Rosenblum and Yom-Tov [5] investigated how people search for information related to attention-deficit/hyperactivity disorder using the Microsoft Bing search engine [10] and Yahoo! Answers, a web QA site. Park et al [6] investigated the use of medical concepts regarding diabetes from the textual data of blogs and QA sites, whereas Yom-Tov and Gabrilovich [7] investigated the side effects of medications from web search queries. Tsuya et al [8] demonstrated that cancer patients share information about their diseases, including diagnosis, symptoms, and treatments via Twitter [11], and Hong [9] explored whether patients could accurately and adequately express their information needs on Chinese health QA websites. Thus, using patients’ claims on the web can provide a qualitative and timely understanding of needs from the patient’s perspective and be considered a type of patient-reported outcome, which may help transform health care in terms of patient-centered care [12,13].

However, there are some limitations to using the accumulated information, the biggest problem being the difficulty in examining a large amount of data. Because there is no existing classification, similar to the CPC, we can only gather a limited amount of information on side effects, for example, by manually processing the data. Therefore, the automatic classification of text data is essential.

### Objectives

This study aimed to extract the needs of patients with breast cancer from the YJQA data and classify them into CPC categories. We adopted the fourth-level CPC categories described above for the classification of patient needs. In the CPC’s first-level categories, the problem granularity is coarse, and it is difficult to understand specific issues. For example, while the CPC's first-level category is outpatient, the corresponding fourth-level categories are “1.1.1.1. Difficulty in obtaining information to select a hospital or doctor” and “1.1.1.2. Difficulty in hospital selection.” Therefore, this study attempted to classify the fourth-level categories to grasp patients’ problems more concretely.

## Methods

### Materials

This study built a data set of 7993 questions submitted to the YJQA between January 1, 2018, and July 31, 2020. The CPC has been systematized to use the problems and burdens of cancer patients, consisting of 16 first-level categories and 631 fourth-level categories. This study utilized 2 corpora: the CPC corpus and the YJQA corpus, for training.

The CPC corpus is a large collection of pairs of cancer survivors’ worries and their labels. The label consists of the CPC category code and the CPC category name (hereafter, both are collectively referred to as CPC categories), obtained from the CPC database [1]. Unless otherwise noted, the CPC categories represent fourth-level categories. An example from the CPC corpus is presented in Textbox 1.

The YJQA corpus is a labeled corpus of 1000 randomly selected questions on breast cancer posted to the YJQA from January 1, 2018, to June 9, 2020. Because multiple different worries are possible, each question is assigned manually to up to 3 different CPC categories. An example from the YJQA corpus is presented in Textbox 2.

**CPC category code, name, and cancer survivors’ worries.**

**CPC category code:** 1.1.1.1

**CPC category name:** Difficulty in obtaining information for selecting hospitals and doctors.

**Cancer survivors’ worries:** I was worried because I had to make decisions based on my limited knowledge and emotions, without any information or indicators to judge whether the hospital's policies and techniques were accurate, especially whether my doctor was trustworthy.

Note: CPC refers to the “cancer problem classification.”

**CPC category code, name, and questions in YJQA.**

**CPC category code:** 1.1.1.1.

**CPC category name:** Difficulty in obtaining information for selecting hospitals and doctors.

**Question in YJQA:** Choosing a hospital for breast cancer treatment. I'm wondering if I'm making a mistake in choosing the first hospital. Is there any problem in choosing the university hospital that is closest to my house?

**CPC category code:** 3.2.2.1./16.3.2.1.

**CPC category name:** I'm worried about finding out the test results/concerns regarding suspicion of cancer (other)

**Question in YJQA:** I had a breast cancer screening and had to be retested for a suspected breast mass. My mother had breast cancer. I will have a mammogram next month. Is the chance of getting breast cancer high? I am very scared and worried.

Note: CPC refers to the “cancer problem classification,” and YJQA refers to Yahoo! Japan’s question and answer service.

We assigned CPC categories to 456 of the 1000 cases, while the remaining 546 cases had no corresponding CPC categories. Thus, the total number of cumulatively classified questions was 661, which were assigned to 133 CPC categories. Table 1 summarizes the most frequent categories, up to the 10th (top 10), regarding the number of questions classified. For example, the most frequent category was “worrying about cancer with subjective symptoms,” with 24.2% of the labeled data falling into this category. Moreover, the category “difficulty in expressing questions and concerns to doctors” was included in the top 10 categories, suggesting that people submitted questions to the YJQA because they had difficulty expressing their concerns to their doctors.

Of the 7993 questions submitted to the YJQA, 6993 were used as the YJQA corpus data classified using CPC categories, excluding the 1000 labeled questions (training data).

**Table 1:** Results of manual classification of YJQA questions (top 10 categories).

CPC category code	CPC category name	n, %
16.3.1.1.	Worrying about cancer with subjective symptoms	160 (24.2)
16.2.1.1.	Matters related to cancer screening	85 (24.2)
12.2.4.1.	Anxiety due to lack of knowledge about cancer	42 (12.9)
16.3.2.1.	Concerns regarding suspicion of cancer (other)	39 (6.4)
9.1.2.2.	Difficulty in asking questions or expressing concerns to the doctor	17 (2.6)
3.2.2.2.	Worrying about the results and their trends	17 (2.6)
12.1.1.1.	Anxiety about the possibility of recurrence or metastasis	14 (2.1)
3.2.1.6.	Concerns about undergoing tests (other)	11 (1.7)
3.1.1.1.	Uncertainty about treatment options	10 (1.5)
3.2.2.3.	Issues related to receiving tests (other)	9 (1.4)

### Classification Algorithm

Our classification algorithm consists of the following steps:

1. Preprocessing: Convert the 2 corpora (CPC corpus and YJQA corpus) into term frequency (TF)-inverse document frequency (IDF)-weighted word vectors.
2. STEP1: Given an unknown problem, convert the problem into TF-IDF-weighted word vectors.
3. STEP2: Classify the target problem into the most relevant CPC category based on cosine similarity between the target problem’s vector from STEP1 and vectors from the 2 corpora.

Here, we extract nouns, verbs, and adjectives using the morphological dictionary mecab–ipadic–NEologd [14] while excluding symbols and numbers. For the TF–IDF calculation, we utilized the TfidfVectorizer under the default parameters in the sklearn.feature_extraction.text module.

Thereafter, we constructed three classification methods using the CPC corpus, the YJQA corpus, and their combined corpus, referred to as the description-based (D-based) method, example-based (E-based) method, and description and example combination-based (D+E-based) methods, respectively.

### Evaluation Methods

We evaluate the accuracy of each method by calculating the proportion of correct classifications. 

The proportion of correct classifications for the D-based method is calculated as follows. First, we find the categories with the highest cosine similarity between the word vectors of the CPC corpus and the manually labeled YJQA corpus (top 1-10). Next, we calculate the proportion of correct categories from 1. Here, it is counted as a correct category if at least 1 of the 3 (maximum) categories is included. Based on the highest cosine similarity, the calculated percentage is referred to as the top 1 accuracy (Acc@1). Similarly, using the top 10 cosine similarities, the top 10 accuracies (Acc@10) are calculated. The proportion of correct classifications is calculated using 5-fold cross-validation to evaluate the E-based method [15]. Using the cosine similarity between the training and validation data sets, the proportion of correct classifications is the mean and median of the rate, as in the above calculation. For the evaluation of the D+E-based method, the proportion of correct classifications is calculated by employing the same evaluation method as for the E-based method using both the CPC and YJQA corpora.

## Results

### Evaluation Results

Table 2 shows the proportions of correct classifications calculated using the above evaluation methods. The Acc@1 and Acc@10 of the D-based method were approximately 10% and 30%, respectively. Furthermore, for both the E-based and D+E-based methods, they were approximately 50% and 70%, respectively. The E-based method is an optimized classification method used to classify YJQA questions. However, it does not cover all CPC categories, whereas the D+E-based method covers all CPC categories, and the rate of correct answers is not significantly different from that of the E-based method. Therefore, in this study, we interpret the results of the D+E-based method.

**Table 2:** Accuracy for each method.

Accuracy			D-based^a^		E-based^b^		D+E-based^c^
**Acc@1**							
	Mean	0.1096		0.4891		0.4781	
	SD^d^	–^e^		0.017		0.018	
	Median	–		0.4835		0.4725	
**Acc@10**							
	Mean	0.2946		0.6960		0.7062	
	SD^d^	–		0.030		0.201	
	Median	–		0.7015		0.7106	

^a^D-based: description-based method.

^b^E-based: example-based method.

^c^D+E-based: description and example combination-based method.

^d^SD: unbiased sample standard deviation.

^e^For the D-based method, there are missing values because 5-fold cross-validation is not utilized as described in the evaluation method section.

### Classification Results

**Figure 1:**
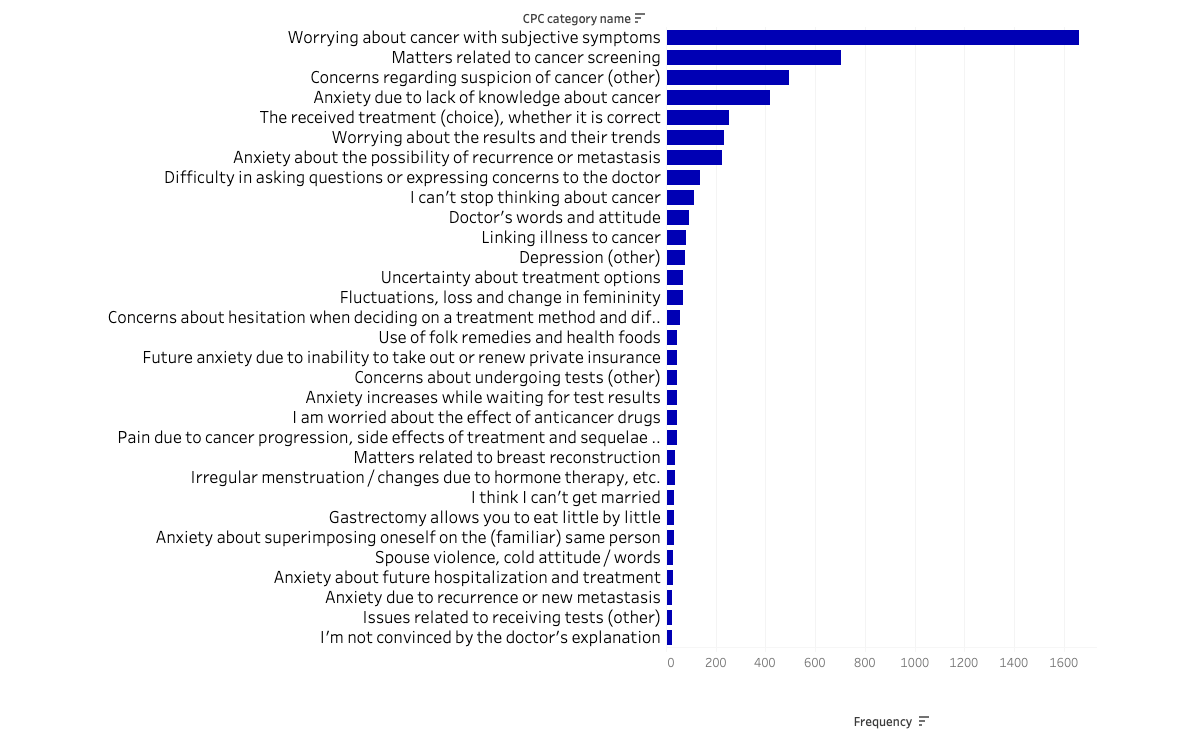
Classification using the D+E-based method (X-axis) and its frequency (Y-axis; top 30 categories). D+E: description and example combination-based method.

We present the classification results of the D+E-based method for the target data to be classified. Table 3 lists the top frequency categories. The top 10 categories accounted for 61.9% of the total. The category with the most frequent questions was “worrying about cancer with subjective symptoms” (1661 questions), which accounted for 23.8% of the total. There were 448 categories classified by the D+E-based method, and the distribution of the top 30 categories is shown in Figure 1. The rate of change from the top 1 to the top 2 categories was the largest at 57.7%. Moreover, the rate of change from the top 20 categories was 20% to 40%, after which it was approximately 10%. As a result, the frequency distribution has a long tail.

**Table 3:** Results obtained using the description and example combination-based method (top 10 categories).

CPC category code	CPC category name	Frequency (%)
16.3.1.1.	Worrying about cancer with subjective symptoms	1661 (23.8)
16.2.1.1.	Matters related to cancer screening	702 (10)
16.3.2.1.	Concerns regarding suspicion of cancer (other)	494 (7.1)
12.2.4.1.	Anxiety due to lack of knowledge about cancer	419 (6)
3.1.3.5.	The received treatment (choice), whether it is correct	255 (3.6)
3.2.2.2.	Worrying about the results and its trend	234 (3.3)
12.1.1.1.	Anxiety about the possibility of recurrence or metastasis	225 (3.2)
9.1.2.2.	Difficulty in asking questions or expressing concerns to the doctor	137 (2)
12.3.2.3.	I can’t stop thinking about cancer	111 (1.6)
9.1.1.1.	Doctor's words and attitude	93 (1.3)

### Similarity of Distribution Between Manual Classification and Our Method

We evaluated whether the frequency distribution of the proposed method was close to that of the real method. Comparing the classification results with the manual classification results (Table 1), we found that 7 out of 10 categories with high frequency were the same, and the first and the second category in both cases were “worrying about cancer with subjective symptoms” and “cancer screening.”

**Figure 2:**
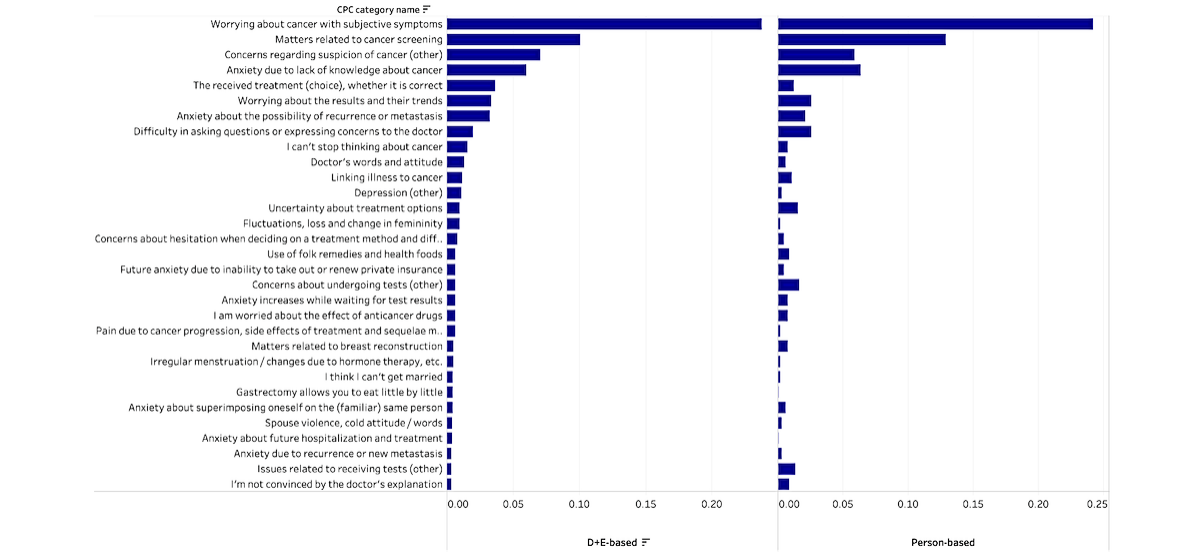
Distribution of the classification results using the D+E-based method and manual classification.

The frequency distribution of the CPC, including the low-frequency part, was compared between the proposed and manual methods. The top 30 categories' frequency distributions in the D+E-based method were used for visual and numerical evaluation of all categories. Figure 2 shows the distribution of the classification results using the D+E-based method and manual classification. The distributions were similar. In addition, we calculated the Jensen-Shannon divergence [16] for all categories to measure the distance between these distributions. Values closer to zero indicated higher degrees of similarity in distribution.

The value of the Jensen-Shannon divergence for the distribution of the manual classification and D+E-based classification result is 0.105, which shows that the 2 distributions are similar. Even though the individual accuracy was low, the CPC distribution obtained by the proposed method was closer to the correct one.

Therefore, it is possible that the proposed method can be used to conduct a large-scale survey of patient concerns automatically.

### Examples

The 3 examples in this section show how the consistency of actual questions and results was confirmed and how the side effects of drugs and unmet needs were extracted.

Table 4 shows the questions estimated to have high cosine similarity. Table 5 shows the questions classified into category 11 (extracted with high cosine similarity) to extract side-effect signaling. In Table 5, we included the code and name of categories classified by our model, drug name, and side effects that could be read from the text of the questions. Table 6 shows some of the questions and their categorization for the low-frequency categories and “COVID” search to extract unmet needs. In Table 6, we included the code and name of categories classified by our model and unmet needs that could be read from the text of the questions.

**Table 4:** Questions estimated to have high cosine similarity.

Questions (translated to English from Japanese)	Cosine similarity	Code and name of categories
Please tell me what makes you susceptible to breast cancer!	0.714	15.1.1.5. I was told that I have cancer
	0.206	12.2.5.1. Suspecting or worrying about another type of cancer
	0.204	11.3.1.1. Current health condition
I had one of my breasts removed due to breast cancer. I did not have simultaneous reconstruction. My breasts are small, to begin with, so when I was asked about simultaneous reconstruction, I didn’t think much about it and told my doctor that I would think about it after the surgery. In my 40s, I was admitted to the hospital, but most people my age had simultaneous reconstruction and expanders. I wondered if I had made the wrong choice. Since If I have to do it later, I'll have to have one more surgery, I think it’s okay as is. I heard that it takes quite a few days to reconstruct. It needs one year at the earliest. Moreover, I heard that nipple and areola surgeries are different. That’s a long time. But I still think I want to have reconstruction. If you have reconstructed, if you haven’t reconstructed, if you have reconstructed in another way, if it’s not covered by insurance, etc, please give me some advice! I’d like to hear about your experiences. It will be two months until my next visit to the hospital. I want to ask my doctor many questions, so if you could tell me anything, I would be very happy. Please give me some advice. Moreover, it seems that the implants and expanders for reconstruction have been discontinued because they are carcinogenic. I don’t think I will be able to have reconstruction for a while, but please advise me.	0.682	13.3.1.6. Matters related to breast reconstruction
	0.264	3.1.1.1. Uncertainty about treatment options
	0.255	3.1.3.5. The received treatment (choice), whether it is correct
I am undergoing treatment for breast cancer, and my white blood cell count has dropped due to side effects, so my immune system is not high. I don’t want to go to the birthday party at my parents-in-law’s because I’m worried that I might get infected with the coronavirus. My mother-in-law and father-in-law know that I am undergoing treatment and my immunity is low, but they don’t want to cancel the party because it’s their adorable grandchild’s birthday. It’s hard for me to tell them. I don’t want my husband to go either, but he doesn’t seem to mind at all. Is there any way to avoid attending the party?	0.657	11.1.2.3. Persistent side effects of anticancer drugs (other)
	0.515	11.1.1.8. Symptoms of side effects from anticancer drugs (other)
	0.417	15.2.16.1. Relationship with family (Other)

**Table 5:** Questions that were classified into the categories of category 11.

Questions (translated to English from Japanese)	CPC^a^ category code and name	Drug name	Side effects
I am undergoing treatment for breast cancer, and my white blood cell count has dropped due to side effects, so my immune system is not high. I don’t want to go to the birthday party at my parents-in-law’s because I’m worried that I might get infected with the coronavirus. My mother-in-law and father-in-law know that I am undergoing treatment and my immunity is low, but they don’t want to cancel the party because it’s their adorable grandchild’s birthday. It’s hard for me to tell them. I don’t want my husband to go either, but he doesn’t seem to mind at all. Is there any way to avoid attending the party?	11.1.2.3. Persistent side effects of anticancer drugs (other)	Anticancer drug for breast cancer	Leukopenia
Can I improve the numbness caused by the side effects of anticancer drug treatment? My sister is undergoing anticancer treatment for breast cancer, and she is suffering from numbness in her hands and feet. Is there anything she can do to relieve the numbness? Does she have to stop the anticancer treatment?	11.1.1.2. Nerve damage such as numbness and discomfort caused by anticancer drugs	Breast cancer drug	Numbness
I am undergoing anticancer treatment, FEC^b^ treatment with infusions every 3 weeks, breast cancer. I have completed four courses, and I am about to start another one, and I have a question about hair loss. My hair still looks like a baby’s, so I can say that I am losing hair. Although I have heard that other parts of my body, such as the eyelashes, eyebrows, shins, and lower hair, I am not losing other than my hair. My doctor said that you lose when I asked my doctor about it the second time. I’m worried that the medication might not be working correctly. If you have any experience with this or know anything about it, please advise me.	11.1.1.1. Hair loss due to anticancer drug treatment	FEC treatment	Hair loss
I would like to know about mouth ulcers during anticancer treatment. I have breast cancer and will start anticancer treatment, but before that, I went to a dentist and was told that I should have my teeth treated. She told me that I would probably get many mouth ulcers from the anticancer treatment but that I should just go and see her. She told me that I should go in. If it’s a common mouth ulcer, I’m sure they can treat it with ointment, but I’m not sure if the mouth ulcer will begin to heal before the anticancer drugs are finished? The side effects of the anticancer medicines make it hard to go to the dentist, and the thought of having to go stresses me out. If I can heal my mouth ulcers faster by going to the dentist, I’ll do my best. However, if it doesn’t make much difference, I don’t want to push myself as much as possible because of the hair loss, fatigue, side effects, and other things. If I go to the hospital because of mouth ulcer, will it heal faster? If you have any experience or know of anyone who had mouth ulcers, please let me know.	11.1.1.6. Mucosal damage caused by anticancer drugs (stomatitis, etc)	Breast cancer anticancer drug	Stomatitis
My 66-year-old mother is undergoing anticancer treatment for the lung’s adenocarcinoma. She is taking Docetaxel plus Cyramza once every four weeks. She had numbness after the second dose and reduced the dose for the third dose, but the numbness keeps getting worse…She’s been taking the maximum daily dose of Lyrica to reduce the numbness, but she says it’s not helping at all. She can’t walk anymore, and it has become mentally painful for her, so we are hoping that we can alleviate her numbness. Can you tell me anything about how to deal with the numbness, herbal medicine, or anything else that might help reduce the numbness a bit? Thank you very much.	11.1.1.2. Nerve damage such as numbness and discomfort caused by anticancer drugs	Docetaxel + Thyramza	Numbness

^a^CPC: Cancer Program Classification.

^b^FEC: fluorouracil, epirubicin, and cyclophosphamide.

**Table 6:** Questions and their classification categories considered as unmet needs.

Questions (translated to English from Japanese)	CPC^a^ category code and name	Unmet needs
My mother has breast cancer with bone metastasis. I heard that bone metastasis has a high risk of fracture, so should I prevent her from driving a car in the future? Moreover, my 80-year-old grandmother is still driving. However, there are many accidents involving the elderly, and the risk of having an accident is probably higher than for younger people. If I assume the worst-case scenario, should I stop her from driving instead of saying, “It’s a pity to take away her car?” If I ask her to quit driving, in what situation/venue should I tell her? Moreover, I have a driver’s license, but I’m a driver on paper only. Should I go back to school to drive for my mother and grandmother when we go out with the family? I don’t think I’ll be able to drive on public roads since I have not driven for a long time…	8.2.1.1. Traffic conditions are bad	Driving a car with a displaced bone cancer patient
Please tell me if I can sue for cancer misdiagnosis. Two years ago, I went to a hospital because a retest was required by mammography. Since there was something suspicious on the echo, I had cytology done on the spot. The cytology didn’t give me any results due to a bad specimen, so I asked for histology. The doctor told me that I would have to stay overnight at another hospital for a mammotome biopsy, etc. I didn’t want to spend a lot of time figuring out what was black and white, so I had a surgical biopsy, a definitive diagnosis that could be done at that hospital. As a result, I was diagnosed with “breast adenopathy” and told to visit the hospital regularly. But some of the results of the tissue examination were not convincing, so I had the examination done at another hospital. The result was breast cancer…how could they remove it from the definitive diagnosis…I would be horrified if they were convinced it was mammary gland disease and discovered it too late. I want to sue the doctor who is still examining and treating me as usual, but I heard that medical lawsuit are difficult. Is it possible to sue him? Do I have a chance to win?	5.3.1.3. Should I get a second opinion?	Lawsuits against misdiagnosis
I had breast cancer sparing surgery in early February and will start radiation treatment in April. However, I am going through a tough time with corona right now, and I feel anxious about going to the hospital every day. Is there anything else I can do except taking personal measures?	8.2.1.3. Frequent visits to the hospital are difficult	Worried about corona infection due to hospital
I had a breast cancer sparing surgery in February this year and was scheduled for radiation therapy, but it has been postponed due to the coronavirus. It will still take some time for the situation to improve, but should I avoid starting radiation therapy at this time? I am on hormone therapy, but I am getting anxious about not undergoing radiation therapy.	11.1.3.6. Symptoms of radiation-related side effects (other)	Treatment postponed due to coronavirus
My 88-year-old mother is in a special care facility and has a fever of 37.5. She has breast cancer, so I don’t know if the fever is caused by breast cancer, corona, or a cold. What are the symptoms of a fever caused by breast cancer? Do I need to see my family doctor? If it is not caused by breast cancer, does the fact that I have a high fever in a special care facility mean that I have contracted the virus from a staff member?	11.2.1.5. Fever	I can’t tell if it’s cancer symptoms or corona symptoms
About 18 years ago, my mother was diagnosed with breast cancer. She had an operation and has been living a normal and healthy life since then. However, 2 years ago, she was told that the cancer had spread to her lungs. At present, she has difficulty breathing even when she moves a little, probably due to the accumulation of pleural effusion. When she was told that the cancer had spread, the doctor did not give her a life expectancy, but when she looked it up on the internet, she found all sorts of information that made her feel uneasy. Can you tell me whether she will live much longer or whether she may be able to live longer while coping with her illness? I’m getting married soon, and I was planning to show her my wedding dress next year. However, with the corona epidemic, that plan is now undecided. I want to show her my wedding dress at least. I’m not sure if this is practically possible.	12.1.1.1. Anxiety about the possibility of recurrence or metastasis	I want my mother to see me in my wedding dress, but she has been diagnosed with cancer
When I distrusted the female surgeon at the [omitted]^b^ Hospital and applied for a second opinion (a letter of introduction was required), I was pressured to go to the hospital for a second opinion. The doctor there is a surgeon famous for his breast-conservation therapy, but he didn’t listen to me very carefully and told me that he agreed with Dr. [omitted] (the doctor in charge at [omitted] Hospital) and that I should tell her that he agreed with her because doctors have a difficult relationship with each other. Is there such a thing? The book on breast cancer published by the [omitted] Hospital, famous for cancer treatment, claims it to be the “standard treatment,” even though the treatment policy is different. Is there anyone who was notified that they had cancer and went for a second opinion and then were offered a different treatment plan? Do doctors always protect their doctors? I was amazed at the lecturers’ pride in the national university hospital (even though they are quacks).	5.3.1.6. Matters related to second opinions (other)	Not fulfilling the role of a second opinion

^a^CPC: Cancer Program Classification.

^b^We blinded the proper noun because it is not relevant to extract the unmet needs.

## Discussion

### Consistency of the Actual Questions and Results

Here we discuss the consistency of the actual questions and results with high and low cosine similarity, respectively. In Table 4, it is unclear whether a cancer patient asked the first question, but it appears to express concern about the possibility of developing cancer. The second question was about breast reconstruction, and the third was a concern about coronavirus (COVID-19).

We also discuss questions that could not be correctly classified in Table 4. The reason for the inability to classify the questions with the highest cosine similarity correctly can be the use of the cosine similarity between the word vectors in the bag of words, and the context could not be taken into account. More specifically, since the question included the word “constitution,” it was considered to be classified in the category that included the word “constitution.” Similarly, the top 2 worries about breast reconstruction could be classified in the CPC category, which includes the phrase “breast reconstruction.” The top 3 problems are related to COVID-19, which is not included in the current CPC category, and therefore must be newly defined.

As for the results with the cosine similarity from the lowest to third-lowest, the question was a request for Japanese translation from English and was not in itself a question about breast cancer. This is because questions including the phrase “breast cancer” were also extracted when searching for “breast cancer,” and the data acquisition method must be improved in the future.

### Clinical Application

In the previous section, we noted that this research was effective for statistical surveys. In addition, we believe that there are other possible applications. In particular, we will examine the extraction of adverse drug events (signal detection) and the extraction of unmet needs.

#### Potential Application to Side Effect Signaling

Extracting side effects from the submitted questions would be very beneficial for pharmaceutical companies and patients because it would allow them to collect significant information on drug safety. Specifically, since some questions classified under the overarching category of “symptoms, side effects, and sequelae” (category 11) of the CPC are considered to contain information on side effects, we can extract such information by applying intrinsic expression extraction to the question text.

In Table 5, the first question contained information about the drop in white blood cells, and the second contained information about numbness in the hands and feet; however, we could not identify the drug that caused the side effect because there was no information about the drug, and the third contained information about the side effects of hair loss due to fluorouracil, epirubicin, and cyclophosphamide (FEC) treatment, a type of chemotherapy. However, the third question simply indicated that FEC treatment caused a side effect called hair loss. Thus, although side effects can be extracted, the granularity of the drug information may be insufficient. Of the 6993 cases, 470 (6.7%) were classified under category 11 using the D+E-based method, of which 100 (21.3%) cases were randomly sampled, and 15 (3.2%) had specific drug names.

#### Potential Application for Unmet Needs

Patients’ unmet needs are becoming a major societal issue. In particular, the unmet needs of those who should answer have not yet been sufficiently addressed. Except for a few fee-based QA sites [17,18], QA sites are generally answered by nonexperts, but some questions should be answered by physicians.

Unmet needs are needs that are not addressed due to a lack of services or resources or that have never existed before. The former may be found by discussing the high-frequency categories with medical workers, which may help identify needs that have been insufficiently addressed in the past, although many patients complain about them. The latter can be extracted by searching for low-frequency categories or words that have become popular in recent years (eg, “COVID”).

In Table 6, the first example is an unmet need (car driving) of a cancer patient with bone metastasis, the second is a misdiagnosis lawsuit, and the third is an unmet need related to COVID-19. In this study, unmet needs were extracted by reading the questionnaire; however, constructing an automatic classification model for unmet needs is a future task.

### Limitations and Future Work

Since we used the Japanese text of questions found by the search phrase “breast cancer” as the training data of the method in this study, the method may not apply to other cancer types and other countries. However, our method can be expanded to different cancer types and countries in cases where problem data are available. Here, the cancer problem categories specific for other countries are needed because they were defined for Japanese people in this study. When expanding our method to other cancer types and countries, future work will have to focus on reproducibility. Therefore, it is necessary to reconstruct the training data from the questions found by searching for each cancer word to apply the method to other cancer types.

In addition, COVID-19 infections in Japan appeared in February 2020, and patients with cancer might experience COVID-19-related problems. Therefore, it is possible that the current CPC categories may not be able to ensure proper classification. Thus, it is necessary to define new problem classification categories for patients with cancer after February 2020. In addition, since new topics, not limited to COVID-19, are always likely to occur, it is necessary to construct a model that could extract such uncommon topics.

The target of this study was question texts posted on QA services, and it may not be possible to classify other texts correctly. The fact that the accuracy of the D-based method was extremely poor among the 3 methods may be due to the difference between the questionnaire text used in the CPC and the text posted on the YJQA. We also found that cancer patients’ problems are not limited to questions posted on the QA website but also Twitter and blogs. It is necessary to broaden the training data of the classification method for these texts to classify the worries of cancer patients. In addition, there are many posts in which the content is unrelated to worries or contains too many emojis. Therefore, it is necessary to build a model to determine whether a post contains worries. Subsequently, 2 schemes are needed to classify the blogs containing worries into CPC categories.

### Conclusions

This paper proposed a method to classify questions submitted to the YJQA into the CPC with a correct answer rate of approximately 70%. Although classification alone does not solve patients’ problems, a comprehensive understanding of the type and number of problems can help prioritize services to solve problems from the patients’ point of view. We would like to examine the services that could be provided in the future based on this information.

## Appendix

Multimedia Appendix 1 Supplementary table containing all the Cancer Problem Clarification (CPC) categories obtained using the D+E-based method.

## Abbreviations

CPC: cancer problem classification
FEC: fluorouracil, epirubicin, cyclophosphamide
QA: questions and answers
TF–IDF: term frequency-inverse document frequency
YJQA: Yahoo! Japan questions and answers
